# The Association between Maxillary Sinus Dimensions and Midface Parameters during Human Postnatal Growth

**DOI:** 10.1155/2018/6391465

**Published:** 2018-05-15

**Authors:** Agnieszka Przystańska, Tomasz Kulczyk, Artur Rewekant, Alicja Sroka, Katarzyna Jończyk-Potoczna, Krzysztof Gawriołek, Agata Czajka-Jakubowska

**Affiliations:** ^1^Department of Oral Rehabilitation, Division of Prosthodontics, Poznań University of Medical Sciences, Poznań, Poland; ^2^Department of Oral Radiology, Poznań University of Medical Sciences, Poznań, Poland; ^3^State University of Applied Sciences, Konin, Poland; ^4^Department of Anatomy, Poznań University of Medical Sciences, Poznań, Poland; ^5^Department of Paediatric Radiology, Poznań University of Medical Sciences, Poznań, Poland

## Abstract

**Objective:**

The aim of the study based on CT images was to assess the age-related changes in maxillary sinus diameters in relation to diameters of the facial skeleton.

**Materials and Methods:**

The retrospective analysis of CT images of the head of 170 patients aged 0–18 years (85 females and 85 males) was performed. Specific orientation points (zy, zm, pr, ns, n, and P) were identified in every patient and the following distances were measured: zy-zy, maximum facial width; zm-zm, midfacial width; n-pr, upper facial height; ns-pr, alveolar facial height; and ns-P, distance not indicated in craniometry.

**Results:**

The maxillary sinuses of every patient were bilaterally measured in three planes. Three diameters were obtained: maximum transverse (horizontal) diameter called MSW, maximum vertical diameter called MSH, and maximum anteroposterior diameter (length) called MSL. In females, the correlation of MSW, MSH, and MSL and zy-zy, as well as n-pr distances, is very strong. Moreover, the significant correlation was found between all measurements of maxillary sinus and ns-pr as well as ns-P distances in females. The correlation between MSL and all measurements of midface as well as MSH and MSW and all measurements except ns-P is stronger in females than in males. In males, all measurements of maxillary sinus correlate with ns-P distance very strongly.

**Conclusions:**

The statistical analysis (correlation and determination coefficient) showed that all measurements of maxillary sinuses correlate with midface dimensions.

## 1. Introduction

Changes in craniofacial morphology observed during the evolution of Hominidae are an important factor that influences the maxillary sinus morphology [1]. The close relation between external cranial dimensions and maxillary sinus volume has been shown in Japanese macaque* (Macaca fuscata)* [2]. Similar correlation, between head circumference and at least two dimensions of maxillary sinus (i.e., vertical and transverse), has been found in the prenatal development of humans [3].

The enlargement of the maxillary sinus is determined by bone remodeling [4, 5]. This process follows resorption of internal walls (except for medial wall) to the extent, minimally exceeding the growth of maxilla. The bone is deposited within the medial wall of the nasal cavity, while simultaneously the lateral wall undergoes resorption. During development, the growth of maxillary sinus jest closely related to the body of the maxilla [6]. In the later period, pneumatisation exceeds the adjacent bones; thus maxillary sinus enlarges at the expense of maxillary processes.

Maxillary sinus pneumatisation is influenced by many factors, that is, teeth development and eruption, maxillary alveolar process pneumatisation, the function of masticatory apparatus, and growth of viscerocranium [7]. Although the presence or absence of maxillary sinus is not dependent on dental morphology [8], the expansion of maxillary sinus can be inhibited by developing permanent teeth [7]. It has been shown that the volume of the maxillary sinus is significantly correlated with environmental factors [9]. Koppe et al. [7] studied the correlation between the depth of maxillary sinus floor and femur head diameter and concluded that maxillary sinus pneumatisation is correlated with the stature. Previously, it has been thought that in Primates the facial dimensions are not correlated with any other body parameters [10].

Tissue morphogenesis of the craniofacial skeleton requires the coordination of a variety of cellular functions to develop complex structures [11]. The process depends on genetic and environmental factors, and any failure or delay in midfacial development may lead to abnormal growth of the orofacial skeleton [12, 13]. During development, skeletal elements of neurocranium and viscerocranium are closely linked with functional spaces (orbits, nasal cavity, and oral cavity) and soft tissues (brain, muscles, and connective tissue) [14]. According to functional matrix theory, structures of head and neck form independent functional units [15, 16]. Every functional unit consists of the functional matrix (soft tissues and spaces) and supporting skeletal unit. According to theoretical assumptions, skeleton of the skull is formed following interrelations between its components, which are controlled by internal factors (hormonal and genetic) [14, 17] and external ones (growth of soft tissues, development of teeth, and biomechanical factors) [14, 17–19]. Enlow* et* Band [4] stated that analysis of the viscerocranium growth as a whole is not adequate because it exhibits different growth patterns of individual functional units. Therefore, in order to speak about the growth processes of the whole viscerocranium, one should analyze separately the growth of individual components of the face. In this context, the analysis of the maxillary sinus growth as separate functional structures seems to be justified.

Despite thorough studies of morphology, dimensions, and volume of the maxillary sinuses in adults, the literature on morphology and growth dynamics of the maxillary sinus in children is vast.

The aim of the study was to investigate the correlation of the maxillary sinus dimensions with the parameters of the midface in children from 0 to 18 years.

## 2. Materials and Methods

### 2.1. CT Scans

The multislice computed tomography (MSCT) scans of patients (aged 0–18 years) from the database of the electronic system (PACS) of the University Children's Clinic were retrospectively studied. All patients who underwent CT imaging of the skull on suspicion of trauma or neurological disease were examined on the 128-slice CT scanner SOMATOM Definition AS+ (manufactured by Siemens) at the Department of Paediatric Radiology. Specimens suffering from neurological diseases or developmental abnormalities, pathologies in the skeletal system, midfacial injuries, or fractures within the skull and paranasal sinus disease were excluded from the study. Scans showing unilateral pathologies within the maxillary sinuses were not included in the study either. Only images described as being normal by radiologists were included in the study.

The access to a hospital database allowed for precise selection of the research sample according to sex and age. The age and sex were found in the medical records; they are also combined with images in DICOM standard.

Finally, the research sample consisted of the CT scans of 170 patients subdivided into 17 groups based on their age. Patients who were 0–2 years old (younger than 24 months of age) were grouped as 1, those who were 2-3 years old (younger than 36 months) as 2, those who were 3-4 years old (younger than 48 months) as 3, and so forth. Finally, the last group, 17, was formed by the patients who were 17-18 years old (younger than 18 years). Within every group, the scans of 10 children (5 males and 5 females) were investigated. A total of 340 maxillary sinuses were examined.

The study protocol was approved by the University Bioethical Committee.

### 2.2. CT Analysis

The linear dimensions of the maxillary sinuses were measured. Slice thickness was 0,5 mm as a standard for further 2D and 3D reconstruction. This allowed reconstruction of volumetric data (3D) on an accuracy level of 1 mm. All evaluations were done using Siemens standard syngo.via workstation (syngo.via software number VD12A), using standard software for image MPR and 3D evaluation.

Measurements were performed on workstation screen with a constant window setting (WL window level 700–600; WW window width 4000–3500) for each measurement.

The metric dimensions were taken by an experienced researcher with the use of a digital marker (caliper) with magnification correction with an accuracy of 0,5 mm. In order to obtain the maximal accuracy and to avoid errors, all measurements were completed three times. Because the differences between the measurements were less than 1%, the mean was calculated and used in statistical analysis.

### 2.3. Midface Measurements

Linear measurements within midface were preceded by identification of the orientation points on CT images according to definitions found in the literature [20–22].

The following points have been designated in every patient:

(i) n (nasion): a point located in the midsagittal plane, on the frontonasal suture, observed on the sagittal image

(ii) ns (nasospinale): a point located in the midsagittal plane, where it crosses the line tangent to the lowermost points of the inferior margins of the piriform aperture, observed in the sagittal image

(iii) pr (prosthion): the most forwarded point of the alveolar process of the maxilla, between the central incisors, observed on the sagittal image

(iv) zy (zygion): the most lateral point of the zygomatic arch, observed in the frontal section

(v) zm (zygomaxillare): the most lateral and inferior point of the maxillozygomatic suture, observed on the frontal image

(vi) P point: determined for the purpose of this study, not defined in craniometry, and is the most distal point of the hard palate, in the midsagittal plane, observed on sagittal image

**Figure 1:**
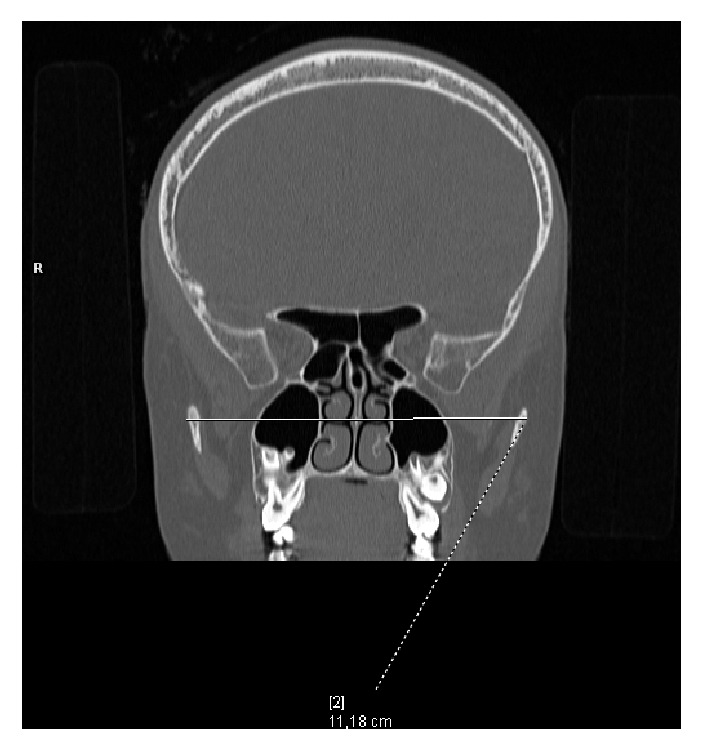
An example of zy/zy distance marked on the CT image.

**Figure 2:**
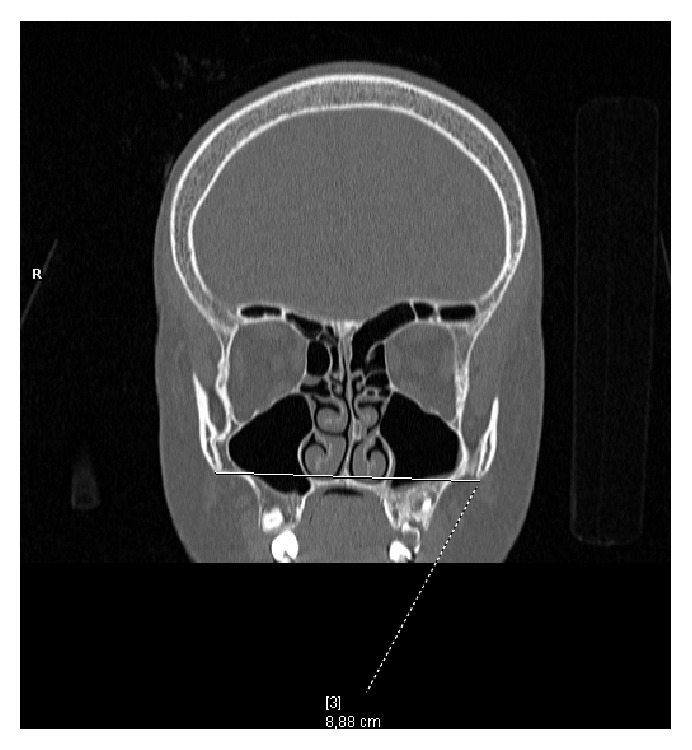
An example of zm/zm distance marked on the CT image.

**Figure 3:**
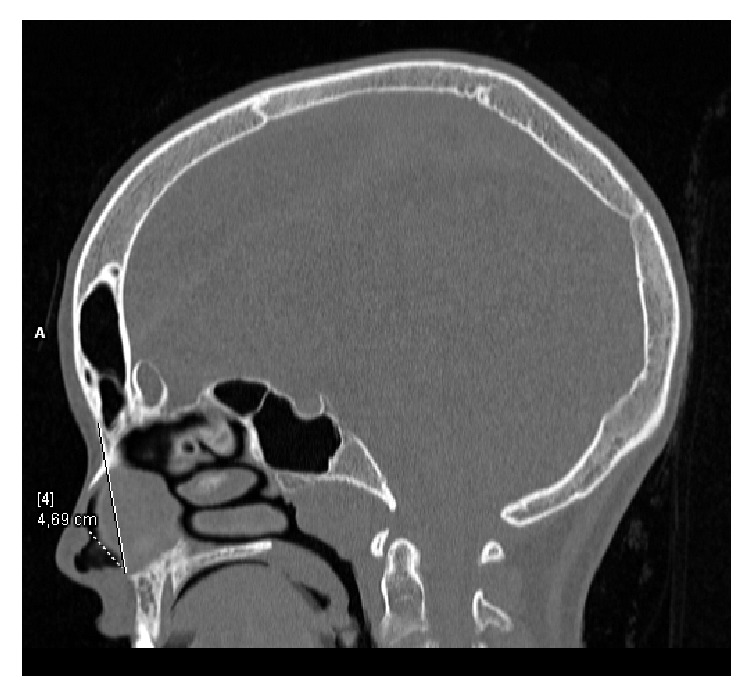
An example of n/ns distance marked on the CT image.

**Figure 4:**
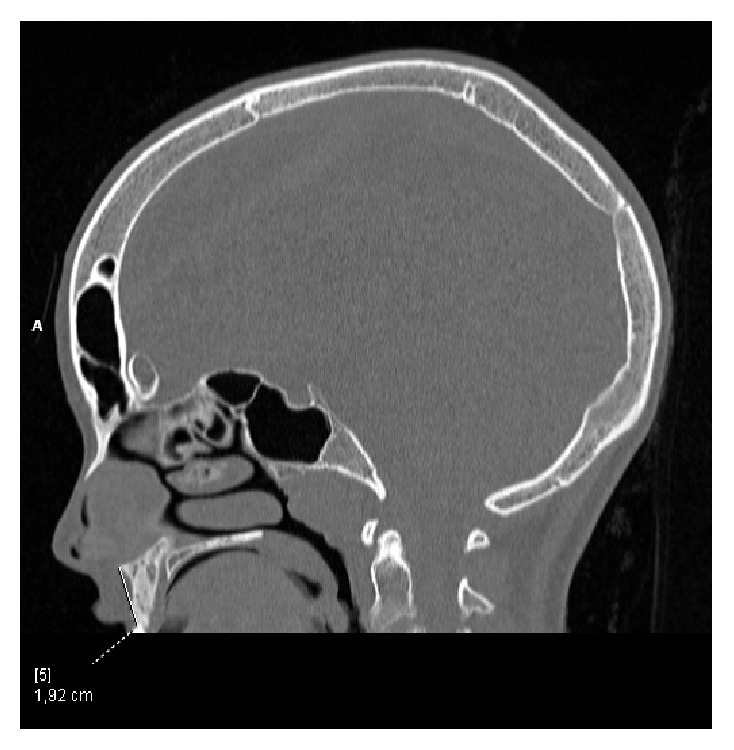
An example of ns/pr distance marked on the CT image.

**Figure 5:**
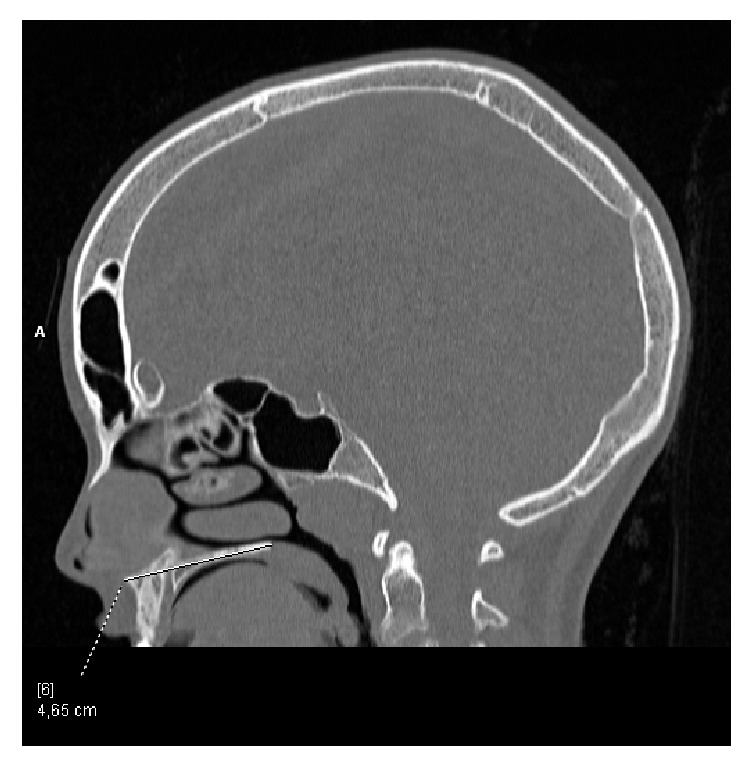
An example of ns-P distance marked on the CT image.

The following measurements were performed (Figures 1–5):

- zy-zy (maximum facial width, interzygomatic facial width)
- zm-zm (maxillary width)
- n-pr (upper facial height)
- ns-pr (alveolomaxillary height)
- ns-P (measurement not found in craniometry)

### 2.4. Maxillary Sinus Measurements

**Figure 6:**
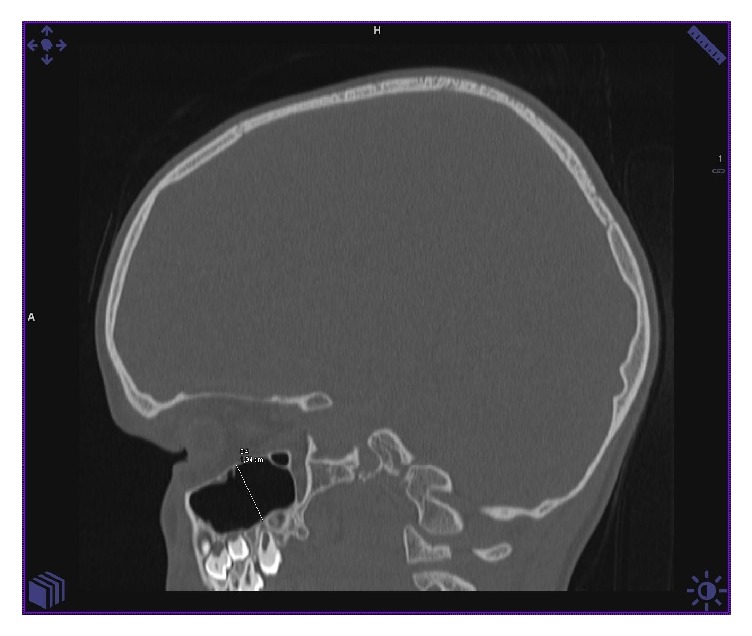
An example of MSH measurement on the CT image.

**Figure 7:**
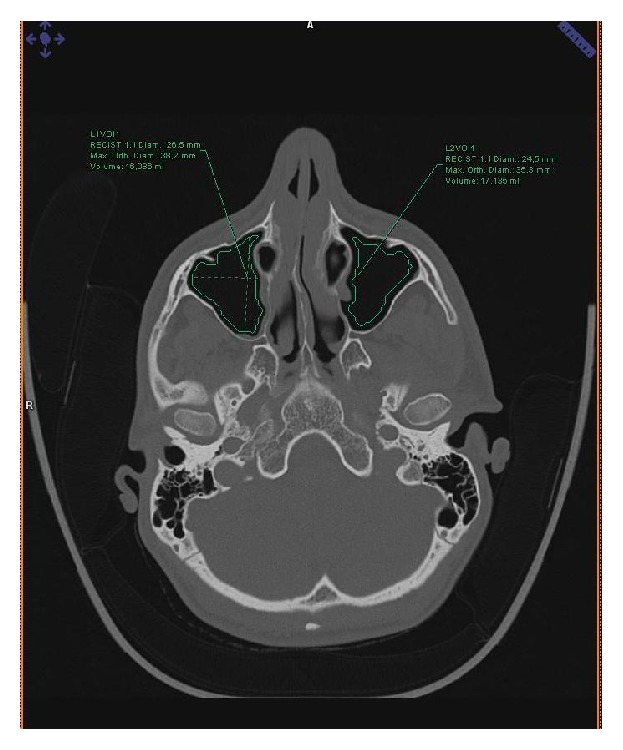
Examples of MSW and MSL measurements on the CT image.

We followed the methods of Lorkiewicz-Muszyńska et al. [6]. Assessment of the maxillary sinus in each patient included bilateral measurements in maximum diameter in three planes (Figures 6 and 7):

(a) Maximal vertical diameter (maximal height) of the maxillary sinus, later called MSH, defined as the longest distance from the lowest point of the inferior wall to the highest point of the superior wall as presented on the sagittal image.

(b) Maximal horizontal diameter (maximal width) of the maxillary sinus, later called MSW, defined as the longest distance perpendicular from the most prominent point of the medial wall to the most prominent point of the lateral wall as presented on the axial image.

(c) Maximal anteroposterior diameter (maximal length) of the maxillary sinus, later called MSL, defined as the longest distance from the most anterior point of the anterior wall to the most posterior point of the posterior wall on the axial image.

### 2.5. Statistics

The statistics were produced by the STATISTICA 10.0 software (StatSoft Inc., USA). The statistical analysis of the data was made by calculating the mean, standard deviation, and standard error, and the Shapiro-Wilk test was used to test the distribution of analyzed variables. The Pearson product-moment correlation coefficient (PPMCC) and coefficient of determination were used to analyze the strength and type of the relationship between variables. The value of Pearson's *r* (between +1 and −1 inclusively) is a measure of the strength of linear dependence between two variables. The closer to −1 or +1 *r* is, the stronger the correlation is.

The coefficient of determination (*r*^2^) gives the proportion of the variance (fluctuation) of one variable that is predictable from the other variable. The verbal description of the relationship between the variables is presented in Table 1.

## 3. Results

**Figure 8:**
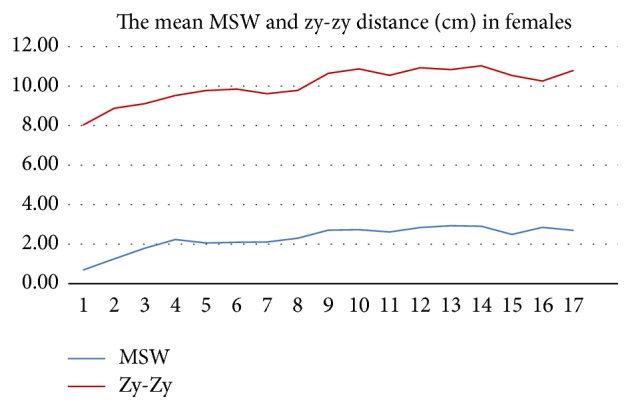


**Figure 9:**
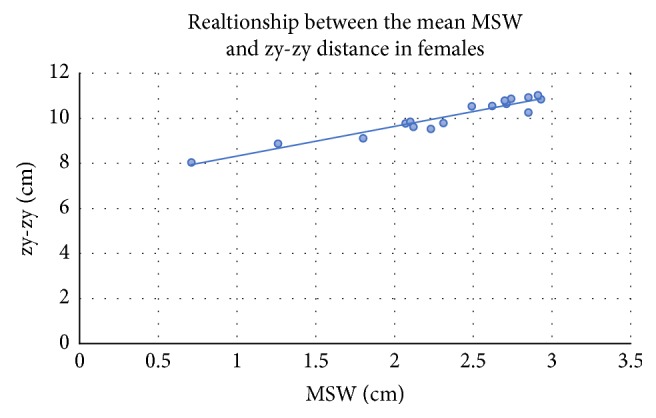


**Figure 10:**
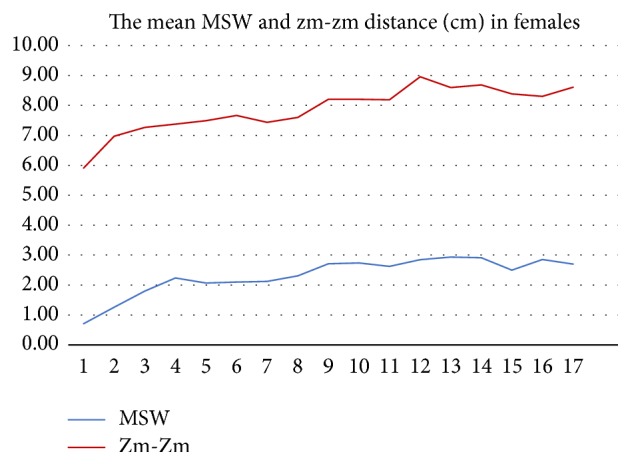


**Figure 11:**
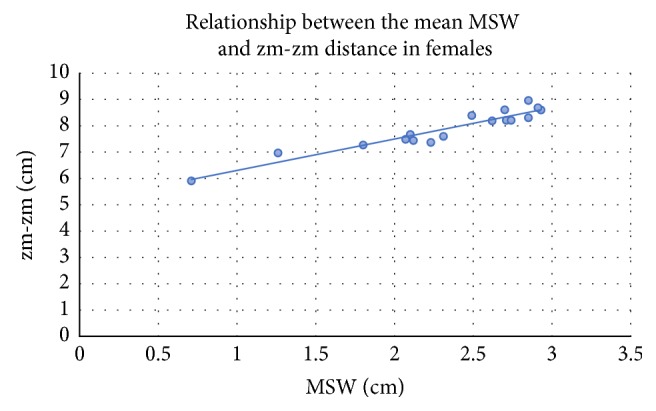


In females, the relationship of MSW with all measurements of the midface was observed. The analysis showed very strong correlation between MSW and both transverse diameters of the midface: zy-zy (*r* = 0,96 and *r*^2^ = 0,92) and zm-zm (*r* = 0,95 and *r*^2^ = 0,90) as well as the n-pr distance (*r* = 0,95 and *r*^2^ = 0,90). A strong relationship between the MSW and the other measurements of midface was observed (Tables 2 and 3 and Figures 8–11).

**Figure 12:**
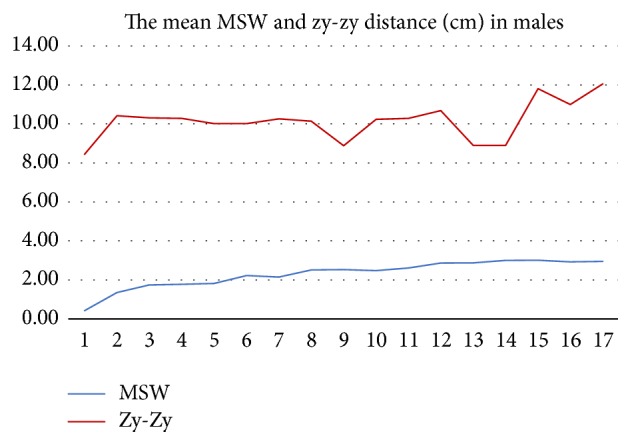


**Figure 13:**
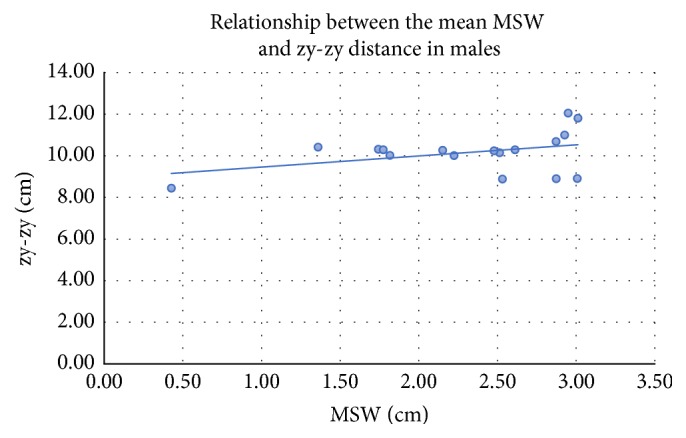


**Figure 14:**
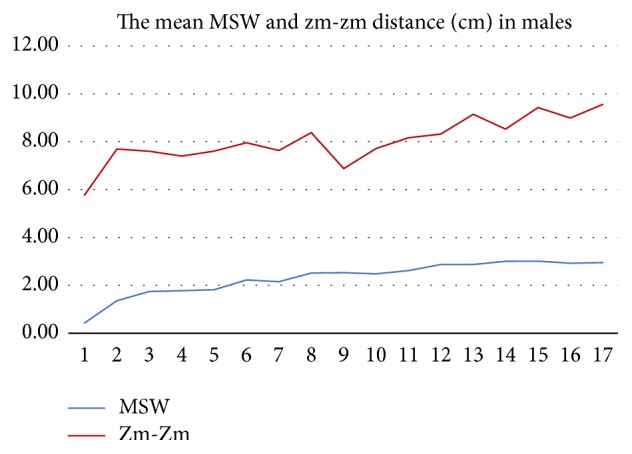


**Figure 15:**
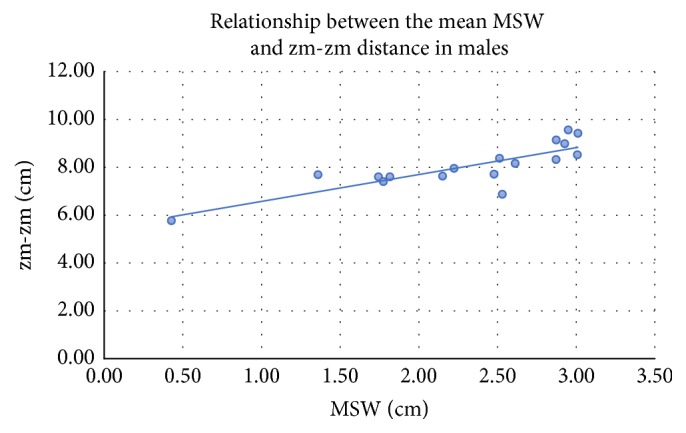


The results in males are different. The MSW shows very strong correlation only with ns-P distance (*r* = 0,94 and *r*^2^ = 0,88). Correlation of the MSW with zm-zm and ns-pr distances is strong, whereas the correlation with ns-pr and zy-zy distances is significant and moderate, respectively (Tables 4 and 5 and Figures 12–15).

**Figure 16:**
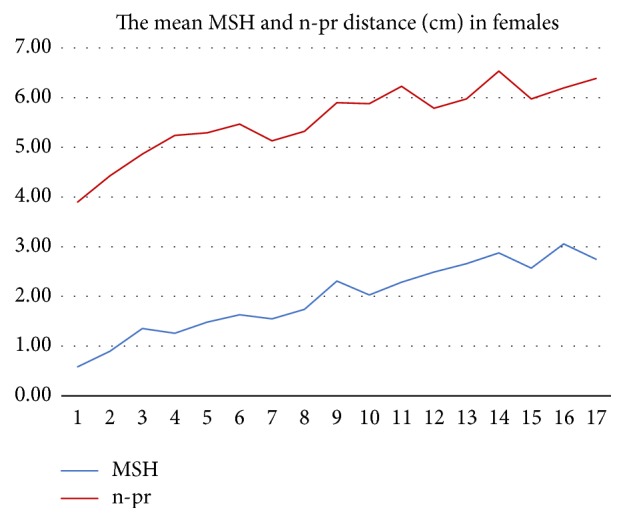


**Figure 17:**
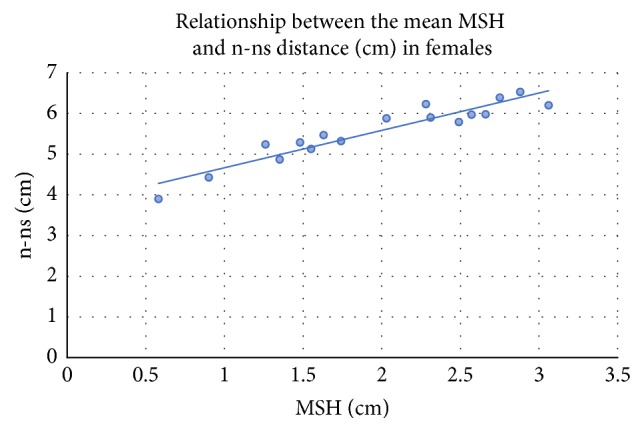


**Figure 18:**
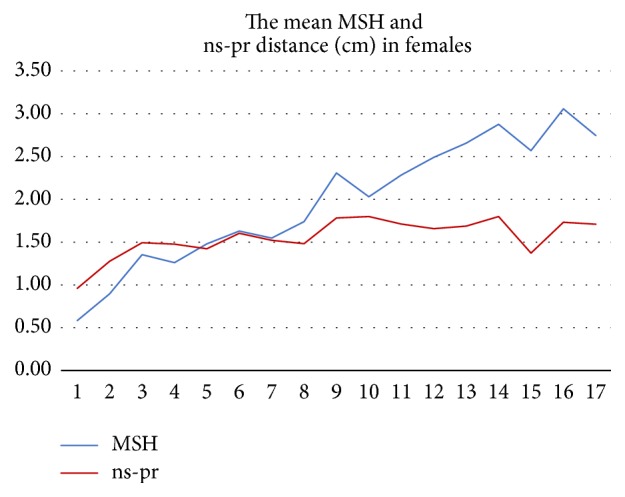


**Figure 19:**
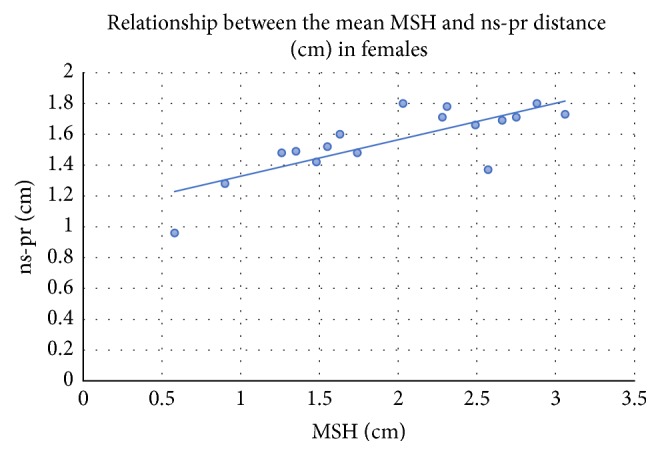


In females, a very strong relationship between MSH and most of distances, zy-zy (*r* = 0.90 and *r*^2^ = 0.81), zm-zm (*r* = 0.93 and *r*^2^ = 0.86), n-pr (*r* = 0.94 and *r*^2^ =0.88), and ns-P (*r* = 0.94 and *r*^2^ = 0.88), was found. A strong correlation between MSH and ns-pr distance was observed (Tables 2 and 3 and Figures 16–19).

**Figure 20:**
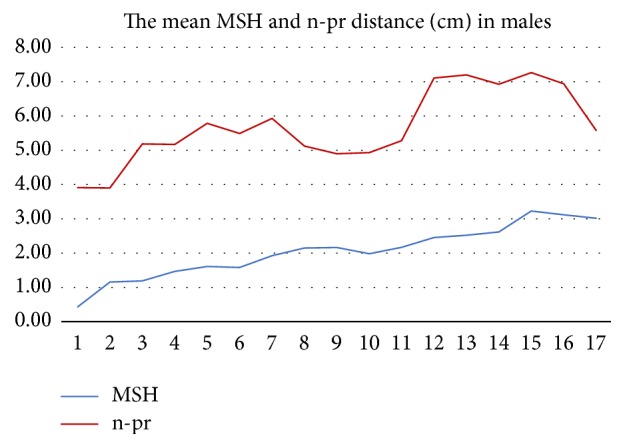


**Figure 21:**
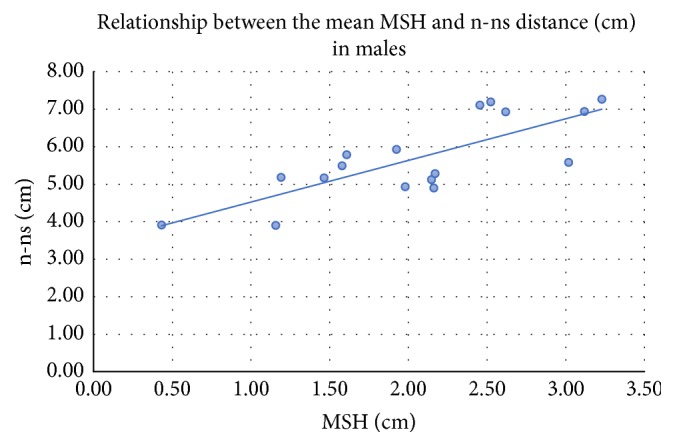


**Figure 22:**
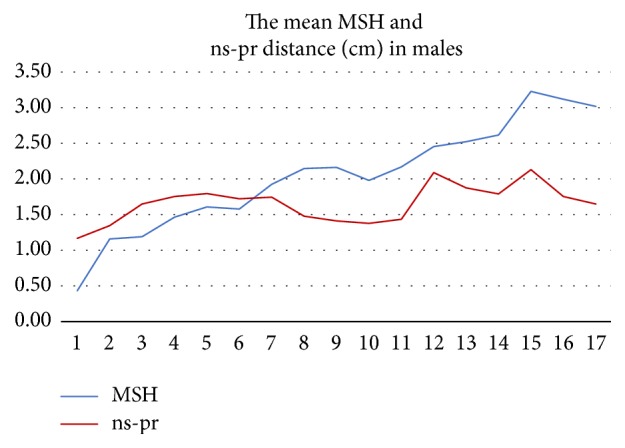


**Figure 23:**
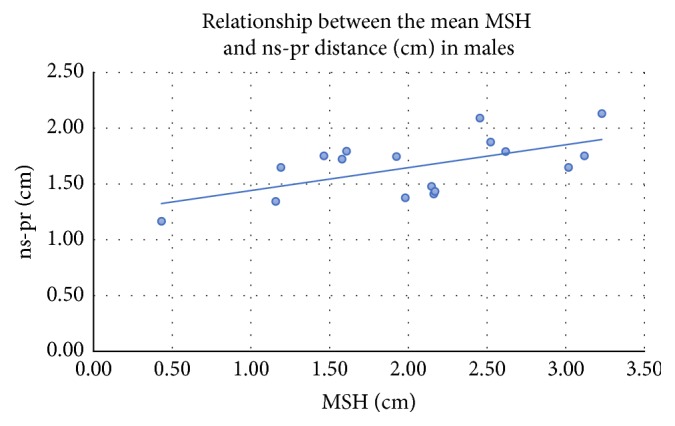


The results for males differ. MSH in males shows very strong correlation only with ns-P distance (*r* = 0.97 and *r*^2^ = 0.94), whereas the relationship between MSH and zm-zm as well as ns-pr distances is strong; it is significant for MSH/ns-pr distance and moderate for MSH/zy-zy distance (Tables 4 and 5 and Figures 20–23).

**Figure 24:**
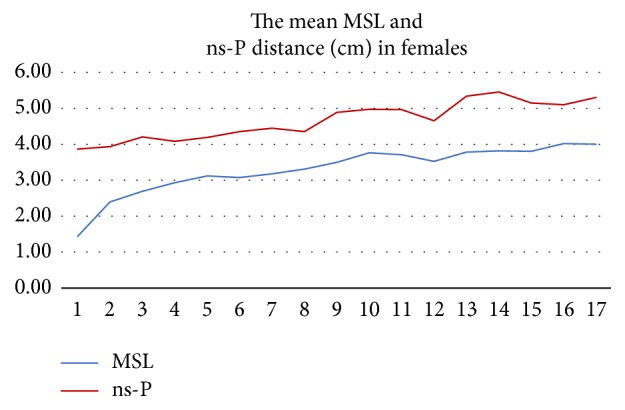


**Figure 25:**
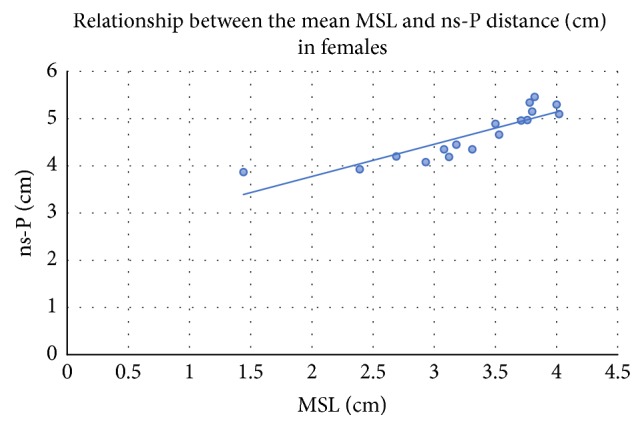


In females, a very strong correlation between MSL and zy-zy distance (*r* = 0.92 and *r*^2^ = 0.85) as well as n-pr distance (*r* = 0.95 and *r*^2^ = 0.90) was observed. A strong relationship between MSL and zm-zm (*r* = 0,90 and *r*^2^ = 0,81) and ns-pr and ns-P distances has been found (Tables 2 and 3 and Figures 24 and 25).

**Figure 26:**
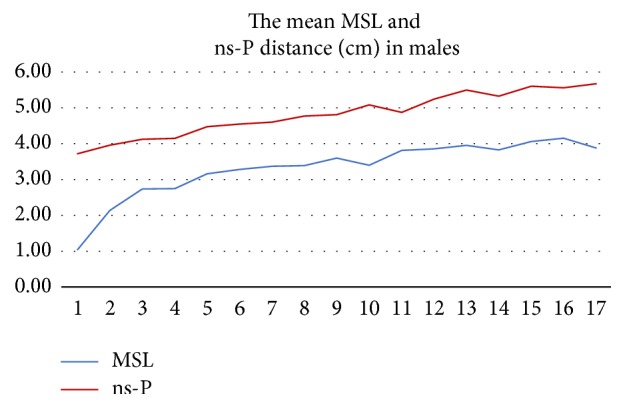


**Figure 27:**
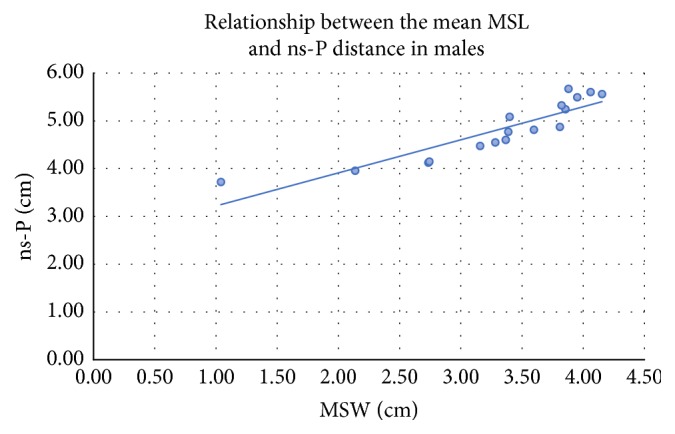


In males, the relationship between MSL and the midface distances differs. MSL shows very strong correlation only with ns-P distance (*r* = 0,94 and *r*^2^ = 0,88), strong correlation with n-pr and zm-zm distances, and significant correlation with ns-pr distance. Correlation between MSL and zy-zy is moderate (Tables 4 and 5 and Figures 26 and 27).

## 4. Discussion

In the presented study, it has been shown that the growth of maxillary sinus distances is relevant to the growth of midface.

Recently, the computed tomography has been more and more useful for descriptive and quantitative analysis of postnatal growth and development of the midfacial structures [23–28].

The postnatal growth of human skull involves dynamic changes in size and shape of viscerocranium. It has been reported that paranasal sinuses parameters are associated with skeletal maturity [29, 30]. Review of the literature revealed no worldwide study on the dynamics of maxillary sinus growth in children in relation to the dimensions of the middle face. Similar studies on frontal sinuses have shown that some dimensions of the frontal sinus are closely related to selected facial features [31].

The pneumatisation of the maxillary sinus is strongly linked with the craniofacial parameters. This correlation has been observed even when severe congenital anomalies exist [14, 32]. Decrease of maxillary sinus volume accompanies maxillary hypoplasia and has been documented in the diseases manifested by developmental anomalies within viscerocranium, that is, Crouzon syndrome, Apert syndrome, Williams syndrome, Goldenhar syndrome, and cleidocranial dysostosis [26, 33–36]. We are convinced that changes of individual dimensions of the maxillary sinus if referred to the appropriate dimensions of the middle face may help to understand the pattern of maxillary sinus growth and the interrelationship between the maxillary sinus and the anatomical facial features.

The results of this study confirm the association between maxillary sinus dimensions and all measurements within the midface in females. Very strong association of all dimensions of the maxillary sinus with distances zy-zy and n-pr was confirmed. The weakest, however, is the relationship of the maxillary sinus dimensions with alveolar height (ns-pr) and the distance ns-P. MSW shows a very strong relationship with the transverse dimensions (zy-zy and zm-zm) and the same strong correlation with the ns-pr distance, which is more surprising. Similarly, MSH has a very strong association with the n-pr distance (vertical dimension) and the same strong association with the distance ns-P (anterior-posterior dimension) and the weakest association with the distance ns-pr. The results of this study confirm the association between maxillary sinus dimensions and all measurements within the midface in females. The relations between all measurements of maxillary sinus and zy-zy and n-pr distances are very strong. The relationship between maxillary sinus diameters and alveolar maxillary height (ns-pr) as well as ns-P distance is the weakest. These regularities apply to all dimensions, not just dimensions in the same plane. As expected, MSW shows a very strong relationship with transverse dimensions (zy-zy and zm-zm). The same strong relationship with the ns-pr distance is surprising. Similarly, MSH has a very strong relationship with the distance of n-pr (vertical dimension), the same strong relationship with the distance ns-P (anterior-posterior dimension), and the weakest relationship with the distance ns-pr.

The weakest relationship between dimensions of maxillary sinuses and ns-pr distance may be due to permanent dentition development. Possibly, the height of maxillary alveolar process affects the growth of maxillary sinus height until the eruption of permanent dentition starts. However, developing roots of permanent teeth influence the alveolar height of maxilla, but they do not contribute to the growth of maxillary sinus height.

The relationship of individual dimensions of the maxillary sinus to the dimensions of the midface of the male is different. MSL correlates with all measured distances in the midface, and MSH and MSW with all but ns-P were lower in boys than in girls. In boys, all dimensions of the maxillary sinus show a very strong association with the distance ns-P.

Curves of all dimensions are very similar. Only in the first two years of life, in both sexes, a more rapid increase in mean MSL than ns-P was observed. The inverse relationship was observed between the MSW and transverse dimensions (zy-zy and zm-zm). Despite the very strong correlation between MSW and dimensions of the middle face in both sexes, it was found that, in the first two years of life, transverse dimensions of the face show a more intense increase than the lateral dimension of the maxillary sinus.

The relationship between MSL and all measured dimensions within the midface and MSH and MSW with all dimensions except ns-P is lower in males than in females. In males, all dimensions of the maxillary sinus show a very strong relationship with the distance ns-P.

Growth curves of all the dimensions are similar. Only during first two years of life, a more rapid increase of mean MSL than ns-P distance was observed in both sexes. A reverse relationship was observed between the MSW and transverse dimensions (zy-zy and zm-zm). In spite of a very strong correlation between the MSW and the transverse dimensions of the midface in both sexes, it was found that, during the first two years of life, transverse dimensions of the face increase more intensively than the transverse dimension of the maxillary sinus.

As demonstrated, the developmental concordance of maxillary sinus and midface dimensions based on correlation coefficients is lower in males (nonetheless statistically significant). This confirms the generally well-known fact that there is gender variation in the characteristics tested and that female gender exhibits greater stability in progressive ontogeny.

The problems presented in this paper do not exhaust the problem connected with the increase of maxillary sinus in the postnatal period. The study value could be increased by extending the size of the study group and including young adults (e.g., up to 25 years). The association of maxillary sinus dimensions with the dimensions of the skull base as well as the dimensions of the other paranasal sinuses can be investigated.

## 5. Conclusions

All measurements of maxillary sinuses correlate with midface dimensions. In females, the correlation of MSW, MSH, and MSL and zy-zy, as well as n-pr distances, is very strong. Moreover, a significant correlation was found between all measurements of maxillary sinus and ns-pr as well as ns-P distances in females. The correlation between MSL and all measurements of midface as well as MSH and MSW and all measurements except ns-P is stronger in females than in males. In males, all measurements of maxillary sinus correlate with ns-P distance very strongly.

## Figures and Tables

**Table 1:** 

*r*	*r* ^2^ 100%	Relationship
≤0,30	≤9%	Weak
0,31–0,50	10–25%	Moderate
0,51–0,70	26–49%	Significant
0,71–0,90	50–81%	Strong
≥0,90	≥82%	Very strong

**Table 2:** The linear correlation coefficient (*r*) for investigated variables in females.

	MSH	MSL	MSW	MSV	zy-zy	zm-zm	n-pr	ns-pr	ns-P
MSH	-								
MSL	0,92	-							
MSW	0,91	0,97	-						
MSV	0,99	0,93	0,90	-					
zy-zy	0,90	0,92	0,96	0,89	-				
zm-zm	0,93	0,90	0,95	0,91	0,97	-			
n-pr	0,94	0,95	0,95	0,93	0,94	0,93	-		
ns-pr	0,78	0,83	0,90	0,78	0,87	0,84	0,87	-	
ns-P	0,94	0,88	0,85	0,95	0,88	0,86	0,92	0,75	-

**Table 3:** The coefficient of determination (*r*^2^) for investigated variables in females.

	MSH	MSL	MSW	MSV	zy-zy	zm-zm	n-pr	ns-pr	ns-P
MSH	-								
MSL	0,85	-							
MSW	0,83	0,94	-						
MSV	0,98	0,86	0,81	-					
zy-zy	0,81	0,85	0,92	0,79	-				
zm-zm	0,86	0,81	0,90	0,83	0,94	-			
n-pr	0,88	0,90	0,90	0,86	0,88	0,86	-		
ns-pr	0,61	0,69	0,81	0,61	0,76	0,71	0,76	-	
ns-P	0,88	0,77	0,72	0,90	0,77	0,74	0,85	0,56	-

**Table 4:** The linear correlation coefficient (*r*) for investigated variables in males.

	MSH	MSL	MSW	MSV	zy-zy	zm-zm	n-pr	ns-pr	ns-P
MSH	-								
MSL	0,99	-							
MSW	0,94	0,91	-						
MSV	0,98	0,90	0,91	-					
zy-zy	0,50	0,49	0,39	0,43	-				
zm-zm	0,87	0,86	0,83	0,84	0,62	-			
n-pr	0,78	0,78	0,77	0,80	0,24	0,72	-		
ns-pr	0,59	0,62	0,59	0,58	0,40	0,63	0,89	-	
ns-P	0,97	0,94	0,94	0,97	0,40	0,85	0,79	0,56	-

**Table 5:** The coefficient of determination (*r*^2^) for investigated variables in males.

	MSH	MSL	MSW	MSV	zy-zy	zm-zm	n-pr	ns-pr	ns-P
MSH	-								
MSL	0,98	-							
MSW	0,88	0,83	-						
MSV	0,96	0,81	0,83	-					
zy-zy	0,25	0,24	0,15	0,18	-				
zm-zm	0,76	0,74	0,69	0,71	0,38	-			
n-pr	0,61	0,61	0,59	0,64	0,06	0,52	-		
ns-pr	0,35	0,38	0,35	0,34	0,16	0,40	0,79	-	
ns-P	0,94	0,88	0,88	0,94	0,16	0,72	0,62	0,31	-
